# Reconciling duty: a theory and typology of professionalism

**DOI:** 10.1038/s41405-023-00172-6

**Published:** 2023-12-04

**Authors:** Andrew Trathen, Sasha Scambler, Jennifer E. Gallagher

**Affiliations:** 1 Consultant in Public Health, London Borough of Hackney, London, UK; 2 Reader in Medical Sociology, Academic Lead for Equality, Diversity and Inclusion, Faculty of Dentistry, Oral and Craniofacial Sciences, King’s College London Sociology and Psychology Office, Floor 18, Tower Wing, Guy’s Campus, London, SE1 9RT London, UK; 3 College Ambassador International, Engagement & Service, Dean for International Affairs, Newland-Pedley Professor of Oral Health Strategy/Hon Consultant in Dental Public Health, Discipline Lead for Dental Public Health, Faculty of Dentistry, Oral & Craniofacial Sciences, King’s College London, Bessemer Rd, SE5 9RS London, UK

**Keywords:** Dentistry, Health care

## Abstract

**Background:**

Professionalism is expected of health professionals and advocated by professional regulators in the United Kingdom (UK). Concepts of professionalism have evolved in sociological discourse and its meaning for dentistry is unclear. It is, none-the-less, considered a core domain of dental education and professional practice by the United Kingdom regulator, the General Dental Council. This paper reports the sense-making process, or social process, of professionalism in practice within England.

**Aim:**

To explore the research question ‘What does dental professionalism mean in practice?

**Methods:**

Taking a constructivist grounded theory approach, involving purposive and theoretical sampling, 24 dental professionals were recruited to participate in this qualitative study. In-depth, semi-structured interviews were conducted by one interviewer (AT). Interviews were recorded, transcribed verbatim, and analysed leading to the development of a theory grounded in the data.

**Results:**

A focus on the social-professional constructs used by participants to make sense of their experiences, resulted in a grounded theory where *Reconciling Duty* emerged as the core category. This represents a process of meeting professional duties to different parties that are often mutually exclusive. It is comprised of three supporting categories: *Applying order to the system*, where individuals attempt to identify what constitutes professional attitudes and behaviours, *Rationalising what is fair*, where individuals make judgements on how the conflict between duties should be resolved, and finally *Responding to the System*, where individuals attempt to actualise these desired resolutions in the context of the complex social system in which they practice. Three dentist archetypes (typologies) emerged, which involved a *personal* (Type 1), *patient* (Type 2), or a *societal* (Type 3) *compromis*e.

**Conclusion:**

Professionalism can be conceptualised as process of reconciling multiple, competing, legitimate duties to different parties, in seeking a fair solution. Once this has been identified, individuals need to work within the complex system of dentistry to make their identified outcome a reality. The findings suggest that using the theory of *Reconciling Duty* helps us to engage with the meaning that the participants drew from the term ‘professionalism’, and anchors it in the lived, everyday professional experiences and challenges faced. A novel typology is proposed, commensurate with calls for a systems approach to the topic.

## Introduction

There is a social expectation that dentists, following our medical counterparts [1, 2], demonstrate professionalism [3]. Although the General Dental Council, the regulator of dentistry in the United Kingdom (UK), places professionalism ‘at the heart’ of their agenda [4], there has been controversy around a shared understanding of the concept. Our preliminary opinion paper in 2009 [5], was at the forefront of a body of research and scholarship exploring what professionalism means in practice for dentistry nationally [6–11]. The UK General Dental Council itself has recently conducted a body of work to inform a shared understanding of what professionalism means in dentistry today [12, 13], which recognises the multifaceted aspects of professionalism in relation to underlying principles and professional behaviours.

In the first half of the 20th Century, sociologists and others were concerned with defining what a ‘profession’ is, and specific traits associated with being a professional. Adopting a functionalist approach, professionalism focused on character traits and virtues which operate at the individual level but have an impact on the social system [14]. This remains the dominant approach in healthcare professions where professionalism as a value system is seen to facilitate interaction with the public [15, 16]. For example, the Royal College of Physicians definition of professionalism incorporates its role as a socially stabilising force, whereby “medical professionalism signifies a set of values, behaviours and relationships that underpins the trust the public has in doctors” [1]. Martimianakis suggests that “a focus on individual characteristics and behaviours alone is insufficient as a basis on which to build further understanding of professionalism and represents a shaky foundation for the development of educational programmes and tools” [17]. Functionalist analyses have generally given way to more critical ideological analyses [18–21].

Scepticism of professionalism as a set of normative values led to a change in the dominant paradigm from ‘structural functionalism’ to ‘interactionism’ in parallel with the changing nature of healthcare including greater use of technology and teamworking. Organisational culture is important [22]; healthcare professionals do not operate in isolation; but, rather, are embedded in systems and environments that provide context for their views and behaviours as reflected in the Royal College of Physicians 2018 publication on ‘Advancing Medical Professionalism’ [23]. Expectations derived from professional frameworks may be ethically ideal, but not necessarily realistic in all circumstances. It is increasingly recognised that the environment can help or hinder individuals in their pursuit of their personal goals, whether that be in motivations, attitudes or behaviours [24–26]. This leads to the question of how organisations may better support professional behaviours, with policy implications for educational and regulatory institutions. Lesser et al. suggest a more useful view may be to see professionalism as the systems of factors that influence professional decision making and behaviour [27]. Proponents of professionalism as a ‘complex adaptive system’ acknowledge that this perspective is in its early stages [28, 29].

Within dentistry, professionalism is one of the four domains of dental education outcomes for the dental team, outlined in ‘Preparing for practice’ in the United Kingdom (UK) [30]. Given its importance in healthcare, together with the emphasis from our regulator, it is therefore vitally important that we consider what professionalism means to members of the profession. Our previous work in this programme of research suggests that unconditional adherence to an externally imposed definition’ [5], does not sit well with the profession [31]. This paper presents the seminal findings of our research exploring what professionalism within dentistry means in practice to those required to demonstrate it. We explore how professionalism manifests itself in their occupational activities, and the nature of the cultural, social, and economic interactions that influence perceptions.

## Methods and methodology

Charmaz, constructivist grounded theory (CGT) [32–34], was employed to recruit participants, conduct and analyse qualitative, depth interviews with dental professionals in England between 2012 and 2017, as outlined by Trathen et al [31]. Ethical approval was granted by the King’s College London Research Ethics Committee (reference BDM/11/12–27, LRS-17/18-5297).

All interview participants worked within the UK dental system, a cross section of National Health service (NHS) primary and secondary care, and private practice. A stratified sample of dental professionals practising around London and the north of England were approached to participate in one-to-one interviews by email. The initial phase of sampling purposively aimed to vary role, practice type (NHS/Private), and patient demographics to represent the different contexts in which dentistry is delivered. Theoretical sampling was employed for later interviews to ensure saturation of categories and develop the emergent theory. Three significant phases of theoretical sampling involved firstly dentists and dental care professionals (DCPs) to explore and contrast perspectives, before refocussing towards private practitioners who had reservations about how NHS care was delivered in the UK. A later phase of theoretical sampling sought dentists who moved away from a primary care environment to explore their reasons for doing so. Participants spanned early (*n* = 9), middle (*n* = 11) and late (*n* = 4) career stages, were mostly dentists (*n* = 22) from primary care (*n* = 20). Informed written consent was obtained for this research and study participants agreed to have their data, including verbatim quotations, used as research leading to publications and presentations.

CGT, as described by Charmaz [32, 34], is well suited for generating novel theory, and ensures it emerges from and remains linked to the data [30]. The constructivist component emphasises the socially constructed frameworks that individuals use to make sense of their lived experiences. Interviews used a topic guide [31], informed by the literature. This was piloted with two respondents and revised in light of the results for subsequent interviews. Initial questions explored respondent understandings of terms used in the RCP definition of professionalism [7]. Interviews were audio recorded with field notes taken, and the data transcribed. Duration ranged between approximately 45–80 mins. Data were recorded and coded using NVivo 10 and Word software.

CGT analysis commenced in parallel with fieldwork as appropriate with grounded theory, with transcriptions undergoing a coding process. The method separated the coding into three fluid stages—initial, focused, and theoretical (Charmaz, 2014). *Initial codes* are descriptive, keeping the content of statements as much as possible while condensing them to their essence. *Focused coding* “requires decisions about which analytic codes make the most analytic sense to categorise your data incisively and completely” (Charmaz, 2014, p.138). They condense a larger amount of text into a concise summary that captures the essence of what the interviewee is saying.

*Theoretical coding* integrates the categories emergent from the focused codes into a wider theory (Charmaz, 2014, pp.150–155). This assembles the categories into a cohesive story and provides direction to the substantive analysis of the earlier stages. A typology [35] emerged from the data and was tested in line with CGT.

At all stages, codes are fluid and change as analysis illuminates established ideas in new ways through the constant comparison process. Memo writing throughout the research helps to record ideas, and they can be expanded and modified as the theory evolves. All these activities occur while data are being collected. The findings are presented below starting with the theory and moving into the typology. All quotations are attributed to participants as follows: designation/male or female/workplace setting/transcription line number (e.g., G1f/NHS/l.100).

## Results

### Reconciling duty: theory structure

The final ‘grounded theory’ consists of a *core category*, *Reconciling Duty,* which captures the overarching social processes at work. This can be broken down into constituent *categories*, which are descriptions of patterns that emerged from the coded data. The categories are theoretically saturated, such that new interviews were not generating novel concepts, and the data generated could be coded and explained by one of the established categories alongside older data with little or no modification to the overall theory.

**Fig. 1:**
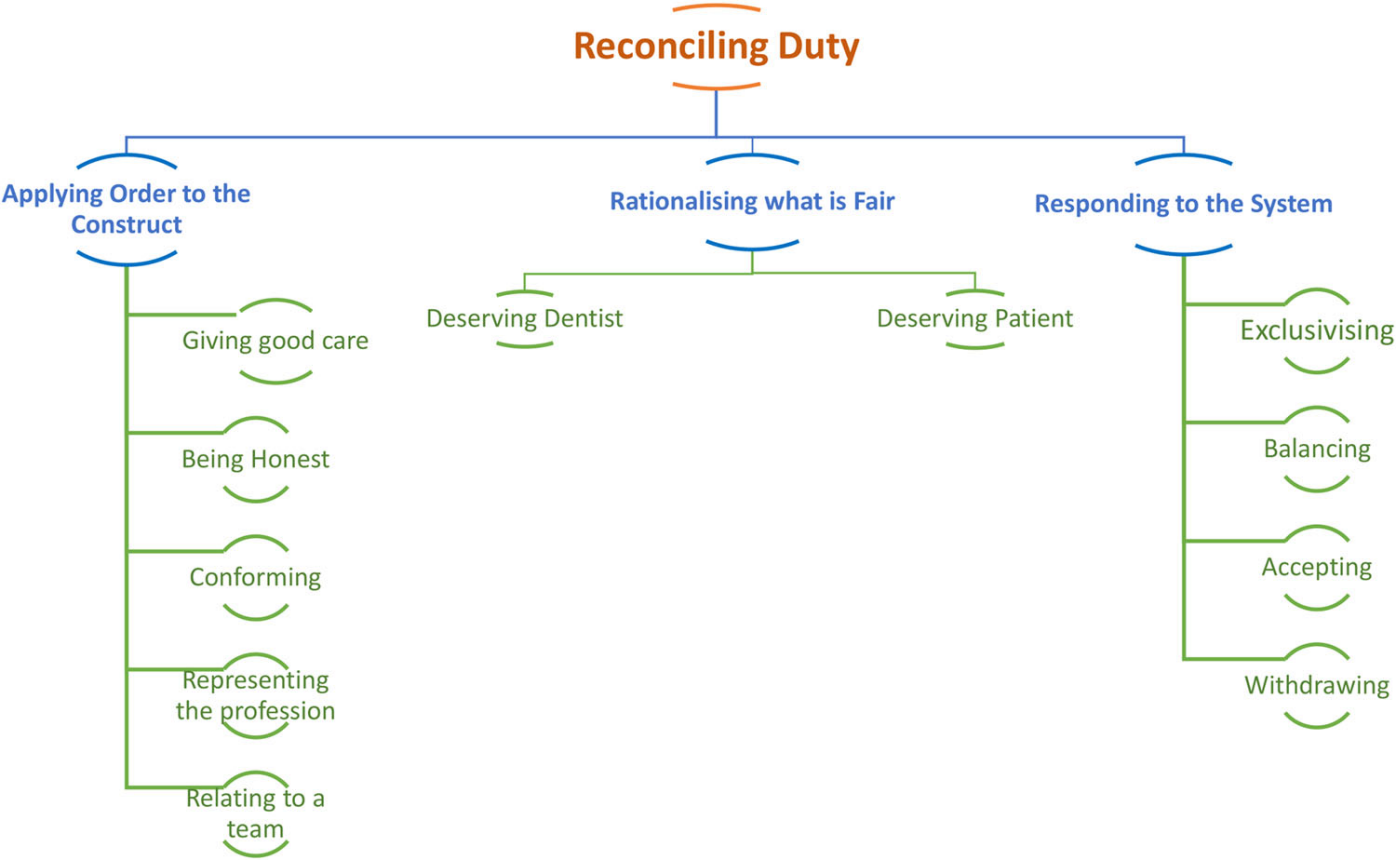
Core category of professionalism: reconciling duty.

The categories contain properties as presented in Fig. 1. These indicate what it is about the category that is relevant and explanatory. Properties are not merely lists of commonly cited codes or concepts but provide a way of understanding the breadth of the category and the variation within it. This variability gives rise to *dimensionality* (Holton, 2010).

Taken together, the *Reconciling Duty* theoretical categories, properties and their variations create a multi-dimensional ‘possibility space’ for the practice of dentistry in its broadest sense. This possibility space is entirely grounded in the data, its topography provided by the dimensions of the properties in each category.

A dentist could occupy any region within this space. However, analysis suggests there are only a limited set of positions a dentist could occupy to maintain a coherent worldview and avoid too much cognitive dissonance. The three most coherent positions are outlined as the final part of this theory as a typology. However, first the theory is presented sequentially below starting with *Applying Order to the System*.

### Category I: applying order to the system

We have made an important conceptual jump in getting close to how our participants acquire meaning from the idea of professionalism. Having shown that collectively, relying on a list of traits leaves us stranded [31], we can get closer to how the participants derived meaning by understanding their social-professional constructs. The construct of each person will be unique, as the messy assemblage of experiences and overlapping worldviews of any given individual will be a microcosm of huge complexity. But it cannot be totally disordered. If it were disordered, then it would not enable people to use it to draw meaning when confronted by an experience or an idea.

The process of achieving order and coherency is captured by the first category of the grounded theory: Applying Order. For this category, the properties are the concepts discussed by participants indicating how they feel things ought to be. There were five properties which captured the range of relevant concepts that emerged from the data: Conforming, relating to a team, representing the profession, being honest, and giving good care as presented below.

#### Conforming

This property captures the feelings that the participants had towards other dentists and whether their behaviour was in line with what the profession ought to look like. There are two types of issues that the participants discussed which involved an expectation of conformity. The participants tended to emphasise one type over the other. The first is an emphasis on the outward facing behaviours, what might be considered the appearance, and the other is an emphasis on attitudes.

*I think the public expect doctors, dentists, lawyers, accountants […], in their professional capacity, to present in a certain manner and I think it gives, especially if you are young, it gives the patients reassurance that you conform to this stereotype in their mind. (G8♀/private/l.117–124)*

Attitudes can be exemplified through language choice. One dentist, for example, related to the way a student spoke to them, which they considered ‘unprofessional’.

*one of them [student] said, “innit” to me…It’s unacceptable…. I said, “what are you up to […] what are you doing, what are you plans for clinic?” “I’m doing MO innit” […] he’s a student and there’s a certain level of respect. I just don’t agree. (G9♀/NHS/l.266–283)*

There are expectations that dentists should look, and behave, in a way that conforms to professional expectations.

#### Relating to a team

This property covered issues where the behaviours and interactions of the dental team were discussed. Some talk focused on behaviours that make teams work well, and it also encompassed leadership styles. There was a clear contrast between those who framed the dentist as the head of the team, and those who had a less hierarchical way of describing things. One participant, for example, talked about attitudes of some of his team members:

*Like attitude for example, I say to my girls, you know what, whatever happens at home, when you come in here […] the patient’s always put first. When a patient walks in I don’t want them to know whether ‘I just had a big breakup with my girlfriend, or whether your ’whatever’ just happened. (G10♂/private/l.311–319)*

Whilst another explicitly highlighted the flat hierarchy within his practice.

*We do believe in listening to all the staff, we discuss anything. Because actually, for me to decide how things should run on reception is a bit silly, because I don’t sit there all the time. So, you know I need the input. Um, so in that sense we’re very flat hierarchical. (G3♂/NHS/l.491–498)*

These quotes both demonstrate attitudes towards the team, their relative positions and perceived expertise, from different perspectives.

#### Representing the profession

This property concerns outward appearances and behaving in a way that ensures that the public perception of dentistry remains good, that the social position of the profession remains high and that dentists are seen to be of good character. This relates to social and professional status:

*I carry the weight of my profession and I have to make sure I keep the, the eminence of my profession at the forefront of my dealing with my patients, that they, they see me as a professional person. (G4♂/NHS/l.158–163)*

And to trust:

*I think you need to understand that you would behave in a way that people would consider appropriate for a professional person, such that the professional impression within the country is such that this is a profession that we can trust and a profession that take their professional responsibilities seriously. (G15♂/NHS/l.1487–1496)*

#### Being honest

There were multiple facets to this property but being honest and having integrity was a common topic and seen as particularly important.

*But you’ve got to be reliable in terms of everything really. You’ve got to be honest. They rely on your honesty, they rely on your professionalism, they rely [on that] you know what you’re doing. So yeah, it’s incredibly important. I think that’s more important than most of them really. (G1♀/NHS/l.739–746)*

Its importance manifested in different ways, such as when dealing with patients, being transparent with fees, being trustworthy, admitting mistakes, and admitting what is available from the NHS.

*Integrity means that you can be honest about what you’re doing and about what you’re charging… And clarity as well about rate charging*.

Can you elaborate on that? (Interviewer)

*Well, you need to be very clear to the patient about what the fees are before they walk in so you’re going to have your fees out there in the public domain. (G11♀/private/l.397–423)*

#### Giving good care

The final property relating to how things ought to be revolves around giving good care. This was important for all respondents. Dimensionality in this property arose from the different facets that were emphasised by different people, such achieving technical quality,

*And I used to like carving amalgams, because I ended up making something tooth-like yeah, it was the sculpture in me, so I felt someone went away, you know, […] they got a quality piece of kit in their mouth. (S1♂/salaried/l.1112–1125)*

Or using quality materials:

*a lot of dentists out there are focusing on getting the cheapest product possible for the patient…. I think this is going to blow up very, very similar to the silicone implant scandal that happened … because the short-term failure is failure of integration of the implant and then there is long-term failure that could be occlusion, it could be peri-implantitis. (G7♂/private/l.426–444)*

Or meeting patient needs:

*The mum kept looking at it, she couldn’t speak much English but kept saying super, super… like she’s really happy. And it’s not like the shade was perfect, you know, contouring was amazing, because even with that he’s moving about, he’s still a child. But he had teeth. That’s enough for me, to be honest. (G1♀/NHS/l.108–115)*

Although it manifested in different ways, the underlying principle of wanting to offer something valuable to patients seemed to be universal. As well as being a property of their process of Applying Order, it provided one of the key motivators for pursuing a career in dentistry. It was important for all the participants to be able to offer something of value.

How did the informants make sense of this value? To whom did they owe it? These questions are answered by the second category, *Rationalising what is Fair*.

### Category II: rationalising what is fair

The fundamental process at work in category II is deciding how the value dentists create through their work is distributed in the fairest possible way. This involves the recognition of different duties. The data showed a perception of explicit duties to patients.

*I think you obviously have a duty of care to your patient and to identify what they want and what they expect and everything (G19♀/NHS/medic/l.571–581)*

There was also a perceived duty to themselves to receive appropriate remuneration,

*Yes, I think that dentists should be very proud of their fees and their income certainly. (G7♂/private/l.567-569)*

Although specifying what this meant in monetary terms was challenging and contextual.

*I have a professional duty to remain viable economically as well. Does that extend to your personal income as well? Well yes because your lifestyle is within … we live in central London; I have four children in school. I’ve got certain amounts of income that I have to take otherwise my kids become homeless and my … it’s all degrees. Yes, I could move to Scotland but if I move to Scotland then I can’t treat my patients in London. So, you have … I have responsibility to the children, I have responsibility to the staff of the practice, I have a responsibility to the patients and that all falls within the economic system. (G11♀/private/l.1393-1410)*

This suggests that there are multiple parties to whom the participants perceived duty, in some form or another. Unfortunately, these duties can often be in conflict. Therefore, the participants needed to rationalise this conflict and arrive at a decision as to what is fair. This does not seem to be a simple or linear process. Sometimes the conflict between duties was recognised as above, but sometimes it was hidden, leading to dissonance.

*Yes, we are in a caring profession aren’t we … “But they can’t afford it?”; “That is not my problem.” That is like going to Sainsbury’s and looking on the shelves at what you can’t afford - does Sainsbury’s care? You know I am not saying leave your patients in pain or anything like that but if they can’t afford their six veneers, they can’t afford their six veneers. That is it, off they go. I can’t afford a new Mercedes, that is how it is. (G8♀/private/l. 809-819)*

Thus far, the categories have described internal, cognitive processes. However, once a person has rationalised what is fair for themselves, their patients, and others around them, they need to find a way of actualising this outcome. All dentists work in a complex system, which may make this challenging. The final category considers the real-world response that allows an individual to bring about what they feel is the fairest outcome.

### Category III: responding to the system

This category focuses on the external environment and systems that influence the practise of dentists in the UK. The system of dentistry places constraints on dentists, making it more challenging for dentists to meet their duties to everyone.

*I think there’s no doubt about it the challenges under the NHS now of meeting NHS targets, balanced again trying to provide high quality ethical modern dental care is getting harder (G15♂/NHS/l.349-353)*

*[Altruism] is incompatible with running a business […] the overheads are massive, the insurances and all of the bits and pieces that come through erm, so we couldn’t, we’d be out of business and if we are out of business, we can’t help anybody. (G6♂/private/l.1438-1442)*

The properties of *Responding to the System* detail how responses allow participants to resolve the challenge of conflicting duties. These properties also help to characterise strategies that are acceptable to some groups of participants, and unacceptable to others. The properties involve accepting, exclusivising, balancing and withdrawing.

#### Accepting

The system of dentistry was perceived as imperfect. Despite great variation in opinion, the NHS was seen to be either flawed, or working in opposition to the interests of both practitioners and patients. Additionally, there was some concern about excessive regulation of healthcare. The first property of *Responding to the System* describes the ways participants reacted towards the flawed system and how easily they could accept it. Some participants were very opposed to the system, and this came through as frustration and sometimes even a palpable anger.

*Sometimes even the paying patients they don’t have the means to pay for the NHS treatment. They’re the ones who get screwed over the most. They’ve just about got a job and they desperately need the treatment but the guy who hasn’t got a job he gets all he wants whereas these guys can’t afford it. (G2♂/NHS/l.43-50)*

The system was considered to have failed to provide affordable care for all, and also to ensure quality, including providing sufficient time to treat patients, and ensure a good work/life balance for staff.

Other participants were also opposed to perceived problems in the system, but they had a more proactive approach; they actively pursued visions for how the system could serve professionals and patients better.

*So, the contract I think needs to work out a mechanism that will enable dentists to be able to continue to work in a more modern, ethical, minimally invasive way but still be able to have business viability because without that you have not got a sustainable system. (G15♂/NHS/l.1056-1077)*

Again, there was acceptance but along with a desire to improve the system.

#### Balancing

The property of balancing describes the activity of acknowledging that it is not viable to offer the best possible service to all the patients who may wish to receive it. Most commonly, this manifested as placing restrictions on the range of services that a dentist would be willing to offer to patients on the NHS.

*I don’t do fixed bridge work on the NHS. Don’t do implants on the NHS. (G9♀/NHS/l.1329)*

These were not merely system limitations, but choices dentists made about which treatments were economically viable to provide. There was also acknowledgement that every person tried to find their balance but that this could be done in different ways.

*I think every dentist tries to find their balance in that system. Whether it be to, whether it be in favour of earning a lot of money and being ethically and slightly morally, um questionable, or whether it be doing a lot of work that perhaps would lose you money, but you’d be able to sleep, it’s essentially, it’s finding a balance between those pressures. (G20♂/NHS/l.690-702)*

The choices made here have a direct impact on both patients and dentists.

#### Exclusiving

This property refers to the decision to achieve a perceived balance of duties by choosing to restrict the care offered to certain groups; specifically, avoiding NHS work, unprofitable patients, or patients who cannot afford services. Carrying out work deemed poorer quality and bringing less remuneration was seen as unfair to themselves and would undermine what it meant to be a professional. Their response was to limit the people who can access their care by practicing privately and setting fees appropriately. This liberates the dentist from NHS constraints and enables them to provide the highest standard of care to all the patients they treat. The extra time is paid for by the patient.

*I guess in this practice I have the luxury of time because I can charge for time, in an NHS practice maybe it’s different, I don’t know. (G11♀/private/l.545-548)*

By limiting, or ‘exclusivising’ the number of people who are eligible to become a patient at their practice no compromise is needed on the quality offered to the patient in front of them; and thus, income and quality are protected.

*I am running a niche private practice I know there are people that can’t afford me, that is fine. You see and I don’t think that makes me any less professional because it is what you do outside dentistry that is where you give back to the society. (G8♀/private/l.1186-1193)*

This property describes the process of balancing the needs of business against the needs of the patient. Dentists could place limits on what they offer patients, setting their own boundaries, and finding an equilibrium that they are happy with.

#### Withdrawing

As the data gathering neared the end stages, and the theory was becoming well established with saturated categories, there remained a final gap which warranted a phase of theoretical sampling to explore the possibility of the reconciling process being too difficult for some people. Given the properties of accepting, balancing, and exclusivising that characterised the category of *Responding to the system*, it seemed quite probable that there would be those who would consider any of these compromises unacceptable or could not neutralise perceived ethical challenges. They may simply withdraw from the system.

Two participants were sought specifically to explore this issue further, one who moved away from primary care dentistry to medicine, and another who was considering career options to overcome these issues. One had lost trust in the dental system and was looking for a career change and the other found a way to provide care without having to reconcile or balance financial issues by retraining as a medic.

*I think that it’s understandable getting a fee for something, I think it was just from my point of view, I always felt like I wanted to be able to help people, that classic cliché, I want to do something to help people, and I felt like I was being made to ask for money where I just didn’t feel like perhaps, I wanted to. (G19♀/NHS/medic/l.77-85)*

For her the financial aspect was a burden that detracted from her desire to help people.

## A grounded theory of reconciling duty

What we can see from our analysis of the data is that *Reconciling Duty* is a process where individuals must necessarily attempt to harmonise multiple, conflicting duties they perceive are being placed upon them, as presented in Fig. 1, to practice successfully. This encapsulates a social process through which decisions are made about which duties are prioritised and how this might be done. The duties identified are not restricted solely to the need to put the patient first, as traditional definitions of professionalism demand, but extend to duties that dentists feel that they have towards themselves, their families, their colleagues and their professional commercial business. When duties to these different parties’ conflict, the tension must be resolved, and the theory of *Reconciling Duty* provides an explanatory framework to understand how the respondents in this study make sense of this tension, and the strategies used to resolve it.

As we have shown, the process of reconciling duties has three stages (categories) to it. Firstly, there is the need to *apply order* to the system. This is the process of understanding the attitudes and behaviours socially expected of a professional within the healthcare systems, and then working out how these attitudes and behaviours fit within the broader social-professional constructs that we use to make sense of the environment and our experiences. The second category involves *rationalising what is fair*. This is the cognitive process of justifying necessary trade-offs when duties to different parties comes into conflict. The third category involves *responding to the system*. This describes the strategies that are adopted to practice in a manner congruent with one’s approach to the reconciliation of competing duties. It necessitates compromise.

All participants demonstrated ways in which they had to make compromises, along with reasons why these compromises were acceptable. Typical compromises might be defending decisions that served business interests, justifying why they chose not to charge a patient for something, explaining why they accepted a reduced income to do some teaching, or why they put limits on the range of care offered to NHS patients.

As the participants described their professional lives and what professionalism meant to them, their social-professional constructs became apparent. They were characterised by reconciliatory behaviours. Regardless of how successful a dentist might be in meeting their duties to some groups, the success requires a trade-off of some kind. They had to make decisions that made sense to them, that fit with their political and cultural worldview, that they believed to be fair and ethical, and that allowed their social-professional construct to be whole and coherent.

## Developing a typology

**Fig. 2:**
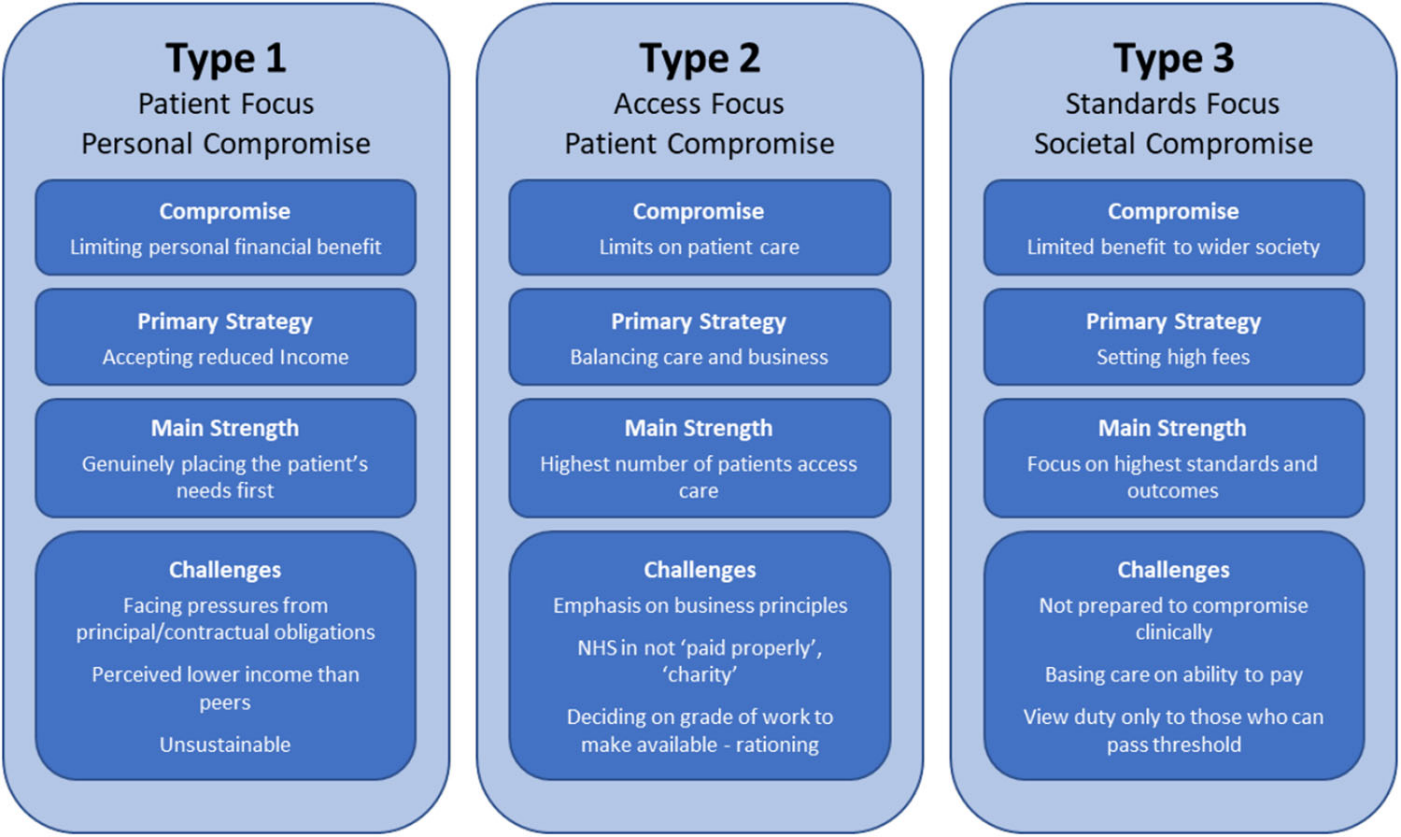
Typology of professionalism in dentistry.

Analysis showed that the process of *Reconciling Duty* consisted of a range of different nuanced positions and viewpoints (dimensionality). This ‘dimensionality’ within the data was important, as it enabled us to identify distinct ‘types’ of respondents. Although a person might occupy any combination of dimensional positions within the theory, we see repeating patterns—some combinations of positions allow a respondent to have a coherent worldview, whereas other combinations would not make internal sense. They would be dissonant. These patterns of coherence allow us to identify three typical ‘types’ of respondent (Fig. 2). When taken as a set, the assembled dimensions of the properties can be triangulated to create a multidimensional possibility space. This possibility space represents all the social-professional constructs that a respondent could conceivably adopt as described through the theory. However, the regions within this ‘possibility space’ that an individual can occupy are limited by the need to be internally coherent and to achieve a stable working situation that allows duties to be reconciled in a balanced way.

It would not be internally consistent, for example, to have a strong belief that everybody is entitled to the highest standard of care, whilst working in environments that permit only more affluent patients to access services. Whilst it is entirely possible to be in this situation, the responses to the system would not be consistent with *Rationalising what is Fair*. There would be a conflict, and the outcome would not be ethically comfortable for the individual. Equally, for an individual who values the highest standard of technical work, it would not be consistent to operate in an environment where the volume of patients and time constraints create a continuous pressure to work faster and compromise on quality.

*And I knew he didn’t have very much money … and I don’t know about him, you know, he might be a sugar daddy behind, his wife might be very wealthy or something like that. But you know, I keep on thinking well you know, the amount of money he’s paying for this, you know, he could probably buy his children a really nice Christmas present or something like that […] But I’ll never be a rich dentist, I like enough to sort of do my hobbies…which are not hugely expensive*,

*(S1♂/salaried/l.869-961)*

The regions of the possibility space that offer the clearest internal consistency within the process of *Reconciling Duty* are presented here as set of archetypes. Of course, nobody’s construct is entirely coherent. In every interview there were logical inconsistencies of which the respondent appeared more, or less, aware. However, there is a distinct logic to each archetype that provides consistency and explains the outcomes of the reconciling process for an individual. The archetypes are not accurate descriptions of any given individual and should not be seen as a rigid categorisation that all dentists will neatly fit into. All the archetypes show great internal variation. However, it is possible to use these archetypes as a model to help us understand the stable and consistent social-professional constructs that explain the reconciliation process outcomes that the respondents struggled towards. This is a conceptual framework to assist in understanding how dentists conceptualise and operationalise professionalism and reconcile their duties as a professional. Three internally logical archetypes were identified as presented in Fig. 2. Each archetype can be characterised by decision to compromise most heavily the needs of one of parties to whom the dentist perceives a duty.

### Type 1—personal compromise

Type 1 dentists compromise on their own earning potential and emphasise an unconditional duty to their patients. Their perspective aligns closely with traditional notions of altruistic professionalism, placing patient needs first and making clinical judgements relatively unclouded by other factors. Of the three archetypes, this is the only traditionally professional group if professionalism is taken to mean, in at least some sense, putting the patient first. The main problem for dentists in this category is the sustainability of their practice. The drive to provide the best for their patients means that they may spend longer with them, use a wider range of materials that are perhaps less cost effective, and do treatments that other dentists might consider charitable in the context of contractual NHS services.

The system of primary care dentistry can be highly problematic for a Type 1 dentist. Their social-professional construct means that they would be uncomfortable limiting or refusing treatment or limiting access to their services. However, the system of dentistry makes this position challenging with the expectations of the practice, the demands of any NHS contract, and the need to make an income that supports them and their family comfortably. There may also be a perception that Type 1 dentists are less successful that their peers. If they do not prioritise income, if they do not seek to move away from NHS dentistry to private practice, if they do not conform to professional norms, then there is a sense of not fitting in with their peer culture. This group struggles to reconcile the competing demands of providing care and making what they do financially sustainable. This relates to a disconnect between a belief that the system exists to help those in need, and the inability for some of the neediest to afford the financial costs that this incurs. Even when a patient can afford fees, Type 1 dentists may be uncomfortable discussing money. It undermines what they feel should be an altruistic activity. For this group, professionalism means putting the patient first and this is incommensurable with making the care they provide a tradable commodity. From this perspective, it is dissonant to call oneself a professional if you are charging for what you do. This is not to say that this group does not recognise the value of what they offer, nor even that they are ethically opposed to charging a fair fee for dental services. But what it does mean is that they are presented with an existential challenge—ensuring the financial success of their endeavours does not easily fit their social-professional construct and reconciliation is difficult.

*I still think the UDA system has failed me as a dentist. In some ways. Some days, some months I’ve struggled to pay rent. Some days I feel that all my efforts have not been, I don’t mean, financially rewarding, but when you’re struggling to pay rent some months. And with the amount that you’re doing for patients, you lose trust in the NHS. You lose trust in the system*.

*(G18 ♂, l.1588-1599)*

*I think that it’s understandable getting a fee for something, I think it was just from my point of view, I always felt like I wanted to be able to help people, that classic cliché. I want to do something to help people, and I felt like I was being made to ask for money where I just didn’t feel like perhaps, I wanted to*.

*(G19 ♀, NHS, l.77-85)*

### Type 2—patient compromise

Type 2 dentists balance the care of their patient with the need to make their practice financially sustainable. A wide range of strategies can potentially be adopted to achieve this including restricting the range of materials available to NHS patients, setting private fees to subsidise NHS care, refusing to offer unprofitable treatments, or limiting the breadth of treatments available on the NHS to those which are most cost effective. The compromise they make is to focus on the patient in the chair they are treating. Although this research does not purport to make numerical extrapolations, the contention is that most dentists in primary care in the UK would operate within a Type 2 archetype, offering a mixture of NHS and private work. The greatest potential benefit from this archetype is improved access for patients across society.

*I am comfortable with managing the low resource of the NHS dentistry to deliver care. If it reaches a stage where I cannot deliver good care I will tell the patient, inform them, ‘This is where […] I can’t go any further because of the economic restraints.’ […] I’ll not compromise*.

*(G4♂/NHS/l.269-276)*

The expectation of seeing high volumes of patients and ensuring they are happy, whilst at the same time carefully managing the resources required to achieve this, allows treatment to occur at scale. It is difficult to conceive how the demand for dentistry could be met without Type 2 dentists, at least within the existing system of dentistry in England. The balance that they achieve is something Type 2 dentists are, for the most part, comfortable with; their balancing strategies permit successful reconciliation of their duties. They treat many people, to the best of their ability, given the economic and systemic restraints they face.

### Type 3—societal compromise

Type 3 would include dentists, who focus on the highest possible technical standards, with low outputs, and commanding high fees. The compromise here comes from limiting access to their services based on ability to pay. Type 3 dentists’ practices are designed to high specifications, and they will typically see far fewer patients than Type 2 dentists. A significant amount of time is spent with the patient throughout the planning and treatment stages. They use the best materials and laboratories, passing costs on to the patients.

*today, I had one, two, three, four, five, six, seven patients, nine wiped out. Each one was anything from, I had two, I had one lady in for two hours this morning, another lady in for two half hours, (with) an hour appointments, (and) half an hour appointments either side - and I am wiped out. Erm and then under the NHS I have seen it. I have seen it, you know it is twenty-five, thirty patients a day, you know*.

*(G7♂/private/l.148-158)*

For dentists working at the most high-end practices, it was recognised that the patients treated would be wealthy. This group of dentists do not routinely treat less affluent members of society. The expectation is that members of the public unable to afford their services should know the fee level before attending. The duty to provide their patients with the highest standard of care is paramount, but poorer patients are unable to qualify as ‘Our Patients’. Type 3 dentists may feel uncomfortable with the idea of operating as Type 2, believing that would entail unacceptable compromises on care or income. Professionally defined standards and quality are most important. The compromise they make is thus societal; some parts of society receive no direct benefit from their professional activity.

*“I used to think ‘…all they are interested in is making money for themselves!’ But when you actually sit down and talk to them you find out that they do so much where they are giving back. But they are not giving it back on the cost of a crown.”*

(G8*♀*, private, mid-career. Lines 1213-1220).

Some informants did not see this as problematic, but there was some evidence of acknowledgement that this approach contravened social moral norms. Even though altruism did not fit happily in their social-professional construct, it was suggested that outside of the working environment altruism was good. The great strength of Type 3 dentists is their commitment to be the best. Although ‘excellence’ was a word that was difficult to interpret for most respondents, for some it was important to excel. Their meaning of professionalism was closely linked to this ideal, achieving the best in terms of technical quality, service, location, and continuous improvement with like-minded peers.

## Discussion

In the context of a complex, messy system of dentistry, this research demonstrates the ways in which participants apply some order that enables them to articulate what dentistry is supposed to look like and how they should practice it. Their fundamental struggle was in determining the most appropriate balance of duties to achieve fairness, and then acting upon that judgement. This is the process of *Reconciling Duty*.

It has been suggested that professionalism in healthcare is too detached from social theory [17]. This study has narrowed the gap by producing a theory that can relate real world practice to the abstract social theories of professionalism. The grounded theory method was particularly suited to addressing the research question [36, 37]. Conversely, those advocating for clear definitions, measurement and assessment [38–40], are also challenged. Professionalism was unanimously deemed important but attempting to formulate a single definition that would resonate with everyone appears to be impossible. It is hard to conceive how a single definition could capture the pluralistic constructs uncovered by this study, as it would inevitably require a normative judgement on what trade-offs are acceptable between the conflicting and mutually exclusive duties that practitioners perceive.

The importance of “Putting the patient first” [41], is conceptually necessary in healthcare, given serious failings in medicine and dentistry over decades [42–44]; and without exception, providing good care to patients was considered very important by participants. Thus, understanding barriers to patient-centric care from the perspective of those delivering it is essential. For the dentists in this study, the realities of dental practice make achieving this goal contingent and conditional. The findings suggest that subtle qualifiers may be added by an individual to make achieving it realistic. The theory of *Reconciling Duty* and associated typology provide a framework to understand the concept of professionalism in practice.

The *Reconciling Duty* theoretical categories create a multi-dimensional ‘possibility space’ for the provision of care. This ‘possibility space’ is entirely grounded in the data, its topography provided by the dimensions of the properties in each category. A dentist could occupy any area or shape within this space, but analysis showed three spaces that were logically consistent—these are the archetypes.

Together, the three archetypes form a typology of social-professional constructs. The conflicts and challenges that each archetype presents are an important part of understanding what the archetypes mean. For Type 1, the challenge is personal, and the respondent must struggle with the consequences of failing to generate the income that their social group would consider acceptable. For Type 2, there is a constant balancing act that must be performed to fulfil their perceived duty to the population of patients who may wish to seek treatment with them, along with the need to ensure their business is sustainable. Type 3 dentists solve this problem of trade-off by ‘exclusivising’ what they do. This leads them to making direct analogies with people who sell commercial products and appears to be associated with political-economic perspectives that maximise the personal liberty to operate and sell their product in a free market.

The problem with the Type 3 archetype is that this excludes potential patients who do not have the economic means to purchase this care, and this exclusion of patients does not fit with some qualities of professionalism as stated by institutions such as the Royal College of Physicians—though it does not contravene any GDC standards. The Type 3 archetype differs from the other two because the data showed some respondents not only describing what they do, but also actively advocating for the benefits of their approach. The Type 3 social-professional construct appeared to be inherently politicised, with evidence of respondents advocating why their perspective leads to better outcomes for everyone. This runs counter to other respondents who valued equity and were uncomfortable charging for treatment. This disconnect is political and profound, and Type 3 dentists who would like to see system change in a neoliberal direction could be considered a counter-cultural movement; it is in direct opposition to calls for professionalism as a defence against commercialism.

Whichever compromise is made, a coherent justification is necessary for a dentist to successfully reconcile their competing duties. The different types allow us to describe and understand how concepts of professionalism manifest for dental professionals. The struggle of a Type 1 dentist can be very hard personally. It seems that it might create a pressure for some to leave primary care dentistry. It is important that this is explored quantitatively. If they are leaving, the collective voice of the profession may become biased towards those who understand professionalism through a Type 3 construct. Type 3 is a natural fit with neoliberal perspectives in healthcare [45]. Debates on health system financing and the commodification of healthcare highlight potential dangers of this approach [45–48].

This study proposes new theory that aids understanding of a topic that has challenged healthcare professionals for decades and has been linked to high profile professional failures intrinsic to public trust. Although it does not settle longstanding problems, it offers new conceptual tools to help understand professionalism in practice. It also demonstrates the value of research from a non-positivist paradigm. In doing so it can present a helpful insight to the research undertaken by the GDC which is more practical than theoretical and avoids a definition, drawing on the work of Welie (2004), and his definition of professionalism as “‘the social contract between the profession and the public (which) entails a collective responsibility of the members of the profession to serve the public good” [49]. Whilst all wished to service the population, the suggested typology helps interpret how, and why dentists may reconcile their duties so differently.

Limitations include selection bias and inaccessible populations. Theoretical sampling was challenging. Identifying individuals with certain views or attitudes carried the risk of making incorrect assumptions; until the interview has taken place, a person’s worldview could not be known. In addition to this, although almost all people contacted in and around London responded to an invitation, arranging interviews further afield was more difficult. This may have been due to greater social distance. Further to this, dentists working at very high-end practices were challenging to contact, and their relatively smaller numbers meant that preserving anonymity was more difficult. However, the sample included a broad range of practitioners with a vast array of differing perspectives and opinions. The theory allows for even more diversity of opinion than captured. This mitigates the bias. However, if quantitative studies based on this theory are developed in future, selection bias would be a potential challenge to external validity.

Typical of qualitative research, the small sample size means that it was not possible to obtain participants that represent the full range of practitioners in England, nor is it possible to make statistical generalisations from the findings. Data saturation is a term not favoured by the authors due to the impossibility of capturing all points of view within a given study, however the major themes became established before completion of the set of 24 interviews. The COREQ criteria were used as the basis for ensuring quality of reporting [28].

Reflexivity refers to the effect that the researcher has on qualitative research, and vice versa. The researcher commences work with a set of ideas, biases, prior interests, and preconceptions. Gibson and Hartman [37], delineate the terms ‘prior interest’ and ‘preconception’. Prior interests are framed as the motivational component for wishing to do research. The researcher would generally attribute some value to the research being done, and it is important to recognise this. Prior interests have the potential to lend strength to the research and analysis and help to make sure the outcome is useful. Preconceptions are ideas and perspectives which we bring to the research and are more problematic as it is often difficult to be fully aware of them. To avoid preconception bias, the research question used no technical language and is kept deliberately open. Similarly, throughout coding technical terms were avoided as much as possible. Theoretical terms were not used until the later stages of the analytic process, ensuring the theory remained grounded in the data rather than being ‘forced’ into any preconceptions of the research team.

The primary researcher for this study was a practicing clinician in general dentistry and continued to practise until the late stages of the project. This provided insight into many specific areas of practice that participants discussed. During the research process it became clear that being a practicing dentist conferred another significant advantage. It allowed for probing and challenging questions as an insider with insider knowledge of the profession. Conversely, being a dentist researcher also introduces biases. It was important not to seek reinforcement of personal views and maintain a critical approach to the participants’ ideas. This was achieved through reflection, discourse and support from the wider research team.

Although there is a substantial body of research literature on professionalism, there is relatively little within dentistry. Research that builds theory resides in the discipline of sociology, and there has been an acknowledged failure to incorporate this in to the work that has been conducted in healthcare [17]. Healthcare research has emphasised either quantitative approaches or qualitative descriptive accounts, predominantly with students in an academic setting, either in the UK or elsewhere [50–55]. This created a large gap to fill, which this single study could not possibly achieve on its own. It, thus, tentatively presents a theoretical foundation for future research, an invitation for further studies complementing or challenging these findings in dentistry and across healthcare, opening allied theoretical avenues, and developing studies to make quantitatively generalisable statements about patients or the workforce.

## Conclusion

Addressing the question “what does dental professionalism mean in practice?”, our findings suggest that professionalism can be conceptualised as process of reconciling multiple, competing, legitimate duties to different parties, in a complex system to achieve a fair solution. Using the theory of *Reconciling Duty* therefore helps us to understand the meaning that the participants drew from the term “professionalism”, and anchors it in the lived, everyday professional experiences and challenges that they face. The theory accounts for the extensive variability in the data and explains the reasoning and actions that dentists make in practice. A novel typology is proposed, commensurate with calls for a systems approach in considering professionalism. These findings have relevance for professional regulators. This research suggests that dentists operate by personal codes of conduct that inevitably come with trade-offs and compromise. Further research may assist regulators in developing a systems-based approach closer aligned to the realities of dental practice.
